# Case Report of Diffuse Large B Cell Lymphoma of Uterine Cervix Treated at a Semiurban Cancer Centre in North India

**DOI:** 10.1155/2016/3042531

**Published:** 2016-08-11

**Authors:** Vibhor Sharma, Tapas Dora, Mehul Patel, Sankalp Sancheti, Epari Sridhar

**Affiliations:** ^1^Department of Medical Oncology, Homi Bhabha Cancer Hospital, Sangrur, Punjab 148001, India; ^2^Department of Radiotherapy, Homi Bhabha Cancer Hospital, Sangrur, Punjab 148001, India; ^3^Department of Radiodiagnosis, Homi Bhabha Cancer Hospital, Sangrur, Punjab 148001, India; ^4^Department of Pathology, Homi Bhabha Cancer Hospital, Sangrur, Punjab 148001, India; ^5^Department of Pathology, Tata Memorial Hospital, Mumbai 400012, India

## Abstract

Lymphoma of the uterine cervix is very rare. We report a case of diffuse large B cell lymphoma (DLBCL) involving the uterine cervix treated at a newly commissioned semiurban cancer centre in north India in 2015. Data for this study was obtained from the hospital electronic medical records and the patient's case file. We also reviewed published case reports of uterine and cervical lymphoma involving forty-one patients. We treated a case of stage IV DLBCL cervix with six cycles of R-CHOP (rituximab, cyclophosphamide, doxorubicin, vincristine, and prednisolone) and intrathecal methotrexate followed by consolidation with radiotherapy. The patient showed complete response to chemotherapy. We conclude that, in advanced stage lymphoma involving uterus and cervix, combination of chemotherapy and radiotherapy is effective in short term.

## 1. Introduction

Non-Hodgkin's Lymphoma (NHL) affects extranodal sites in one-third of cases. The most commonly affected extranodal sites are the gastrointestinal tract and skin. Rarely may female reproductive organs be involved, most commonly ovary. NHL of the cervix is extremely rare. In one series, cervix was involved in 1 out of 730 cases of NHL and 1 out of 175 cases of extranodal lymphoma [1]. It is usually a high grade B cell lymphoma [2] with abnormal vaginal bleeding as the most common presenting symptom (60%) [3]. As cervical lymphomas arise from the stroma rather than the mucosa, hence cervical cytology is not very sensitive in recognizing it. We report a case of DLBCL involving uterine cervix and provide a review of literature of cervical lymphoma.

## 2. Case Presentation

**Figure 1:**
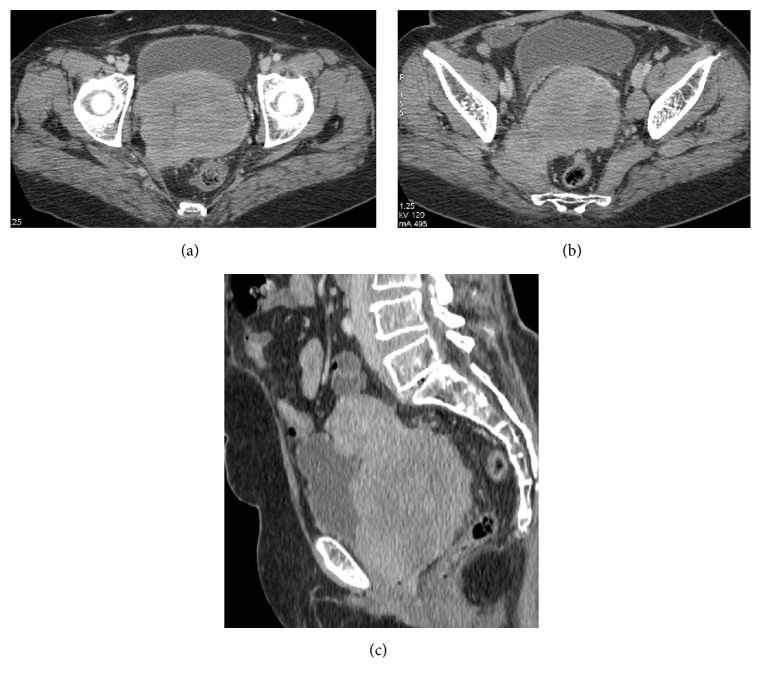
Prechemotherapy scan shows that an ill-defined enhancing lesion is seen involving the cervix invading the bilateral parametria (a) and uterus (c) and also extending into the right mesorectal fascia and piriformis muscle (b).

**Figure 2:**
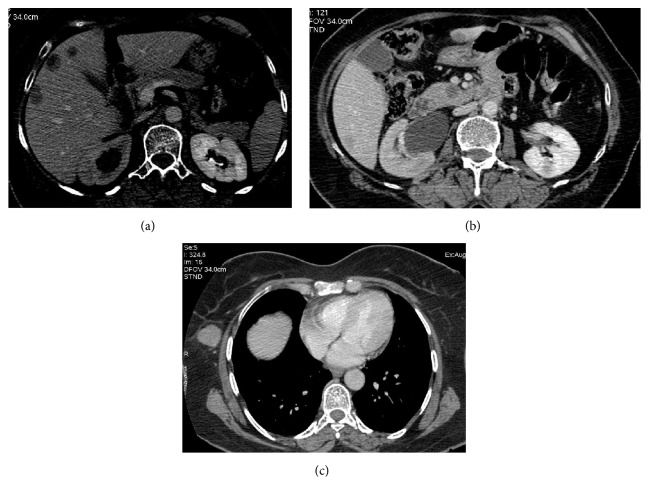
Prechemotherapy CT scan showing multiple ill-defined hypodense lesions in liver (a) and hypodense lesion in the uncinate process of pancreas (b) and in the right chest wall (c). Moderate hydronephrosis on right side secondary to lower ureteric involvement by the cervical mass (b).

**Figure 3:**
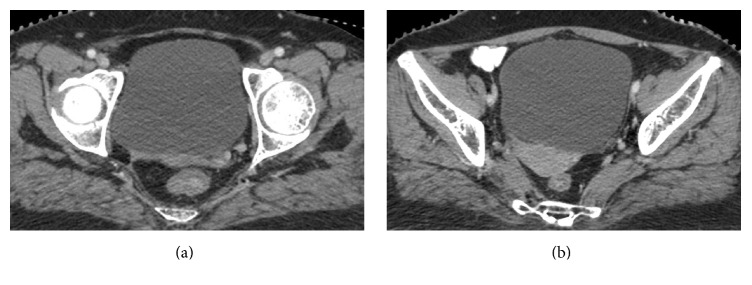
CT scan after six cycles of chemotherapy showing almost complete regression of the cervical mass and its extensions (a, b).

**Figure 4:**
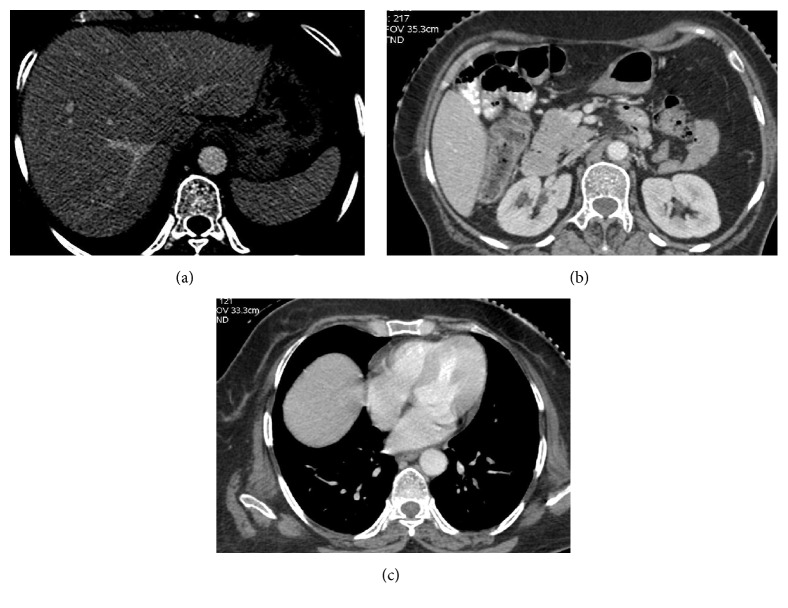
CT scan after six cycles of chemotherapy reveals complete resolution of the liver, pancreatic, and chest wall lesions.

A 61-year-old postmenopausal lady presented with bleeding per vaginum and 6 kg weight loss in preceding 3 months. Her performance status was 1 (ECOG scale). Local examination revealed a 7 × 6 cm mass in the cervix and extending to lower uterus and upper third of vagina, involving both parametria. Rectal mucosa was uninvolved. There was no hepatosplenomegaly or lymphadenopathy. Contrast Enhanced Computed Tomography (CECT) scan of chest abdomen and pelvis revealed a 8.7 × 7.9 cm mass in cervix with extension into uterus and lower cervix, abutting the urinary bladder, laterally involving parametrium, encasing right ureter causing mild-to-moderate hydronephrosis and posteriorly loss of fat planes with rectum; bilateral external iliac lymph nodes were enlarged (largest 2.5 × 2 cm); 1.3 cm mildly enhancing mass in uncinate process of pancreas; multiple hypodense lesions in both lobes of liver, largest being 1.5 cm; and 2.8 × 2.4 cm soft tissue deposit in subcutaneous plane in right lower chest wall and multiple subcentimetric nodules in bilateral lungs (Figures 1 and 2). Bone marrow and cerebrospinal fluid were uninvolved. Cervical biopsy revealed diffuse large B cell lymphoma. On immunohistochemistry, cells tested positive for CD20, MUM 1, and BCL 6; Mib 1 proliferation index was 70–80%. Viral serology for HIV, Hepatitis B surface antigen, and Hepatitis C were negative. After complete diagnostic workup, she was diagnosed as DLBCL stage IV BEX IPI 3/5. She was treated with 6 cycles of 3-weekly R-CHOP (rituximab, cyclophosphamide, doxorubicin, vincristine, and prednisolone) and intrathecal methotrexate 12 mg on day 1 of each cycle. After four cycles, response assessment CT scan showed almost total resolution of all lesions. After 6 cycles of chemotherapy, contrast enhanced CT scan showed complete response (CR) (Figures 3 and 4). After completion of the planned chemotherapy, she received involved field radiotherapy to cervix 45 Gy/25 fractions over 5 weeks. She completed her treatment in January 2016. During treatment she developed grade 1 peripheral neuropathy.

## 3. Discussion

NHL of the uterine cervix is a rare tumour. The most common symptoms are vaginal bleeding, vaginal discharge, and pelvic discomfort and dyspareunia [2]. Differential diagnoses of cervical lymphomas include sarcoma, poorly differentiated carcinoma, neuroendocrine tumours, malignant mixed Mullerian tumour, melanoma, extraosseous Ewing's sarcoma, and chronic cervicitis. Most cases of NHL involving uterine cervix are stage I or II. The optimal treatment of such tumours is not clear. These tumours have been managed with chemotherapy, radiotherapy, and surgery [4–6], alone or in combination. In a study of twenty-six cases of NHL involving the uterus [3], there were ten cases of stage I E or II E primarily involving the uterus and twelve cases of stage III E/IV involving the uterus. The median age of presentation in the two groups was 55 years and 58 years, respectively. DLBCL accounted for 80% and 50% of cases in the two groups, respectively, the remainder being constituted by follicular lymphoma, small lymphocytic lymphoma, marginal zone B cell lymphoma, and precursor T cell lymphoblastic lymphoma. The five-year survival rates in the two groups were 83% and 29%, respectively [3]. Various chemotherapy regimens have been used for DLBCL of cervix. These include MACOP-B [2] (methotrexate, cytarabine, cyclophosphamide, vincristine, prednisolone, and bleomycin), CHOP [2, 4, 7] (cyclophosphamide, doxorubicin, vincristine, and prednisolone), BACOD [4] (bleomycin, doxorubicin, cyclophosphamide, and vincristine), R-CHOP [8–10] (rituximab, cyclophosphamide, doxorubicin, vincristine, and prednisolone), CVP [11] (cyclophosphamide, vincristine, and prednisolone), CHOP-bleo [12] (cyclophosphamide, doxorubicin, vincristine, prednisolone, and bleomycin), ASAP [12] (doxorubicin, methylprednisolone, cytarabine, and cisplatin), and CEOP [13] (cyclophosphamide, etoposide, vincristine, and prednisolone). These patients attain prolonged disease-free survival with combination of chemotherapy and radiotherapy.

## 4. Conclusion

NHL of uterine cervix is a rare tumour. It may be primary or secondary. It is usually high grade B cell lymphoma. Deep biopsy is required for diagnosis as surface cytology is frequently negative. Standard treatment is not established. In advanced stage disease, combination of chemotherapy and radiotherapy is the treatment of choice with encouraging early outcomes. This treatment is feasible in semiurban settings in developing countries.
